# The strong emergence of molecular structure

**DOI:** 10.1007/s13194-020-00308-7

**Published:** 2020-10-01

**Authors:** Vanessa A. Seifert

**Affiliations:** University of Bristol, Bristol, UK

**Keywords:** Strong emergence, Molecular structure, Downward causation

## Abstract

One of the most plausible and widely discussed examples of strong emergence is molecular structure. The only detailed account of it, which has been very influential, is due to Robin Hendry and is formulated in terms of downward causation. This paper explains Hendry’s account of the strong emergence of molecular structure and argues that it is coherent only if one assumes a diachronic reflexive notion of downward causation. However, in the context of this notion of downward causation, the strong emergence of molecular structure faces three challenges that have not been met and which have so far remained unnoticed. First, the putative empirical evidence presented for the strong emergence of molecular structure equally undermines supervenience, which is one of the main tenets of strong emergence. Secondly, it is ambiguous how the assumption of determinate nuclear positions is invoked for the support of strong emergence, as the role of this assumption in Hendry’s argument can be interpreted in more than one way. Lastly, there are understandings of causation which render the postulation of a downward causal relation between a molecule’s structure and its quantum mechanical entities, untenable.

## Introduction

Emergence is a topic which raises heated debates in philosophy. The term ‘emergence’ is used in philosophy of science in order to describe a relation either between scientific theories, descriptions, or representations (this is usually labelled ‘epistemic’ or ‘weak’ emergence), or between entities, properties, etc. that occupy at least partially distinct levels or scales of ontology (this is usually labelled ‘ontological’, ‘metaphysical’, or ‘strong’ emergence).^1^ While the distinction between epistemic and metaphysical emergence is to an extent accepted in the literature, any further attempt to specify emergence falls short of wide agreement.^2^

There are various disputes about how to formulate emergence in the literature. For example, philosophical accounts often specify emergence by reference to sets of terms that are at least partially distinct (for example Silberstein 2002: 90–92; Humphreys 2016: 38–39; Wilson 2015: 364–372). This makes it difficult to compare alternative accounts of emergence (Kim 2006: 548). Another disagreement lies in the specification of the relata that participate in the emergent relation. For example, regarding metaphysical emergence, philosophers speak of emergence between parts and wholes, properties, events and processes, causal capacities, laws, entities (Silberstein 2002: 90), features (Wilson 2015: 373), or phenomena (Batterman 2011: 1031). Most commonly, philosophers use some set of these notions interchangeably, without implying a change in content (for example Wilson 2015: 349). There are, however, accounts of emergence that disagree on this point (for example Batterman 2011: 1031). Another disagreement concerns the appropriate criteria for emergence. For example, some accounts take ‘explanatory irreducibility’ to be sufficient for emergence (Bedau 2002: 48; Kim 1999: 21); other criteria include epistemic/conceptual novelty (Butterfield 2011; Franklin and Knox 2018), robustness (Franklin and Knox 2018), the irreducibility of theories, and unpredictability (Batterman 2011: 1031).^3^

In response to this divergence, some philosophers propose a unified understanding of emergence either by arguing that there is a specific set of notions that underwrite all accounts, or by showing how all accounts are compatible with their proposed one (for example Wilson 2015: 348; Gillett 2016: 359). This paper does not follow any of these strategies in order to examine emergence. Instead, it examines one of the most detailed putative case studies of strong emergence that is available in the current literature, namely Robin Hendry’s account of the strong emergence of molecular structure. Obviously this is an incomplete analysis of emergence, but this is unavoidable in light of the disagreements that exist in the literature. Nevertheless, the critical examination of Hendry’s account contributes to the philosophical discussion about strong emergence.

Hendry argues that the structure of a molecule strongly emerges from its quantum mechanical entities. He characterises this with reference to downward causation, according to which, ‘the emergent behaviour of complex systems must be viewed as determining, but not being fully determined by, the behaviour of their constituent parts’ (Hendry 2006: 180). Section 2 presents strong emergence and defines downward causation (DC), as this is argued for by Hendry with respect to molecular structure. It examines downward causation in the context of Kim’s distinctions between diachronic and synchronic, and between reflexive and non-reflexive DC, and argues that only reflexive diachronic DC is plausible for molecular structure. Section 3 presents the putative empirical evidence that supports the strong emergence of molecular structure. Section 4 argues that there are three challenges that have so far remained unnoticed and that undermine the viability of Hendry’s account of strong emergence.^4^

## The strong emergence of molecular structure

There are three core elements to Hendry’s understanding of strong emergence. These are:

(i) A hierarchy of levels;
(ii) the existence of a dependence relation; and,
(iii) the causal autonomy of the higher-level.^5^

(i) suggests that the entities postulated by the examined pair of theories are found at different levels of ontology. As Humphreys states, ‘there is an ontology that is divided into an ordered hierarchy of levels, with the more fundamental entities occurring toward the bottom of that hierarchy’ (2016: 8). Whether the postulation of a hierarchy of levels is necessary for the strong emergence of molecular structure, is not examined here; it is taken for granted as being one of the core elements of Hendry’s account.^6^ Moreover, a discussion about how ‘levels’ should be understood isn’t pursued here.^7^ This paper takes ‘levels’ to refer to distinct energy, length, and/or time scales.^8^ In this context, nature is divided into distinct scales of ontology that the physical and special science theories specify by postulating entities, properties, etc. that are found at distinct (yet possibly overlapping) energy, length, and/or time scales.
(ii) postulates a dependence relation between higher and lower-level properties. While there are alternative understandings of this dependence relation, Hendry understands it in terms of supervenience (Hendry 2010b: 185). Applied to chemical properties, supervenience postulates the existence of a relation between chemical and quantum mechanical properties, such that when any chemical property of a molecule changes, then it is always the case that one or more of its quantum mechanical properties have changed as well (Hendry 1999: 120).
(iii) postulates the causal autonomy of the higher level. The higher level is causally autonomous in the sense that there is some set of higher-level entities, properties, etc. that possess causal powers which are distinct from the powers possessed by the lower-level entities, etc. on which the former depend (i.e. supervene, in accordance with clause ii).^9^ These powers are distinct in the sense that they are not token identical to any set of powers possessed by the lower-level entities, etc. (Wilson 2015: 362).

There are various ways in which one can further specify the causal autonomy of the higher level (Wilson 2015: 347). This paper examines Hendry’s understanding of causal autonomy (and thus of strong emergence), which is specified in terms of downward causation. That is, the higher level is causally autonomous iff it exhibits downward causal powers.

Hendry defines strong emergence in terms of downward causation as follows:

the emergentist sees (special-science properties) as distinct and non-reducible just because the causal powers they confer are not exhausted by those conferred by their physical bases. The additional causal powers are exerted in downward causation.(..) Emergentism invokes downward causation- the special-science properties sometimes push their physical supervenience bases around. (2010b: 185)

Hendry’s understanding of DC involves the claim that the higher-level causal powers produce effects at the lower level. Hendry does not explicitly state what notion of causation he has in mind with reference to DC. He proposes a ‘nomological formulation of emergentism’ which suggests that DC is at least supported (if not understood) in terms of nomological sufficiency (2010a: 218). On the other hand, Hendry examines DC with respect to Kim’s examination of the overdetermination problem and of the CCP. Kim’s analysis is based on an understanding of ‘(c)ausation as generation, or effective production and determination’ (Kim 2005: 18) and he does not provide a more detailed understanding of causation as production (see also List and Menzies 2007). Given this, this paper formulates DC in terms of Kim’s notion of production as a primitive (without further clarifying this notion), and in subsection 4.3 it examines how Hendry supports DC in terms of nomological sufficiency.

Moreover, Hendry does not specify whether the higher-level causal powers that participate in the downward causal relation, are necessary or sufficient to produce lower-level effects. If the higher-level causal powers are necessary, then this doesn’t exclude lower-level causal powers in participating in the determination of lower-level effects. If they are sufficient, then this means that the higher-level causal powers determine certain lower-level effects without the involvement of lower-level causal powers. The ambiguity of this aspect of DC is a weakness of Hendry’s account because depending on this, the requirements for downward causation are different. For example, subsection 4.3 examines whether it is tenable to consider higher-level causal powers as being sufficient in determining lower-level effects and argues that it is not.

Lastly, Hendry investigates DC with respect to Kim’s distinctions between synchronic and diachronic DC, and between reflexive and non-reflexive DC (Kim 1999; Hendry 2010b: 188–189). These distinctions result in four alternative notions of downward causation: reflexive synchronic, non-reflexive synchronic, reflexive diachronic, and non-reflexive diachronic DC. It is not entirely clear which of the four notions Hendry assumes with respect to molecular structure. While he accepts Kim’s objections against a synchronic notion, it is not clear whether he supports a reflexive or non-reflexive understanding of diachronic DC (Hendry 2010b: 189). This ambiguity is revealed in the passage where Hendry contrasts the case of a molecule’s structure with the case of a falling vase. He states that there is ‘a clear difference’ between the two cases, ‘whether the molecule’s causal powers are exerted internally or externally (that is, *reflexively or non-reflexively*)’ (Hendry 2010b: 189).^10^

Due to this ambiguity, the remainder of this paper examines strong emergence in the context of all four notions of DC. It argues that only diachronic reflexive DC is coherent and applicable to the case of molecular structure. However, in the context of the latter notion, there are three challenges that undermine the tenability of the strong emergence of molecular structure.

### Reflexive synchronic DC

This subsection argues that reflexive synchronic DC should be rejected because synchronicity is incoherent given the ‘causal-power actuality principle’ (Kim 1999: 29) and supervenience.

Reflexive synchronic DC, as a form of strong emergence of molecular structure, is stated as follows. At t, there is at least one non-empty set of token causal powers:

- possessed by the chemical entities which define system W’s structure (i.e. the structure of a molecule);
- which is not token identical to any set of causal powers possessed by the quantum mechanical entities of W; and,
- which produces certain aspects of the quantum mechanical entities of system W.

Two objections against reflexive synchronic DC are considered here. First, following Kim, synchronic reflexive DC goes against what he defines as the ‘causal-powers actuality principle’. The principle states that:

for an object, x, to exercise, at time t, the causal/determinative powers it has in virtue of having property P, x must *already* possess P at t. When x is caused to acquire P at t, it does not already possess P at t and is not capable of exercising the causal/determinative powers inherent in P. (Kim 1999: 29)

In the case under examination, this means that, if the chemical entities that specify the molecule’s structure possess token causal powers that produce certain of the quantum mechanical properties, then certain properties of the quantum mechanical entities do not yet exist at that moment. This means that DC cannot be a synchronic causal relation between the token causal powers of the chemical entities of a molecule and the molecule’s quantum mechanical ones. Therefore, the notion of reflexive synchronic DC is incoherent. Further support to Kim’s ‘causal-powers actuality principle’ is not provided here, and in any case, Hendry accepts this as a plausible principle and objection to DC (Hendry 2010b: 189).

Secondly, if supervenience is assumed, then the incoherence of reflexive synchronic DC is revealed even without appeal to Kim’s previous objection and the ‘causal-powers actuality principle’. Supervenience is a synchronic dependence relation between the lower-level and higher-level properties of a system. If the higher-level properties determine some set of lower-level properties, then this set does not yet exist at t. However, synchronic DC supposes that the supervening chemical properties exist at t, since it claims that certain of its token causal powers produce quantum mechanical properties at t. Therefore, we arrive at the incoherent result that the supervenient chemical properties exist at t, whereas the complete set of the respective properties on which they supervene is not instantiated at t. Thus, supervenience cannot hold at t. This is contradictory to one of the core elements of Hendry’s understanding of strong emergence, namely that supervenience holds at t.

### Non-reflexive diachronic & non-reflexive synchronic DC

This subsection examines both non-reflexive diachronic and non-reflexive synchronic DC since both notions are rejected on the same grounds.

Non-reflexive diachronic DC, as a form of strong emergence of molecular structure, is stated as follows. There is at least one non-empty set of token causal powers:

- possessed by the chemical entities, etc. which define system W ‘s structure at t_1_;
- which is not token identical to any set of causal powers possessed by the quantum mechanical entities, etc. of W at t_1_; and,
- which produces certain aspects of the quantum mechanical entities, etc. of system S (distinct from W) at t_2_.

Non-reflexive synchronic DC, as a form of strong emergence of molecular structure, is stated as follows. At t_1_, there is at least one non-empty set of token causal powers:

- possessed by the chemical entities, etc. which define system W ‘s structure (i.e. the structure of a molecule);
- which is not token identical to any set of causal powers possessed by the quantum mechanical entities of W; and,
- which produces (at t_1_) certain aspects of the quantum mechanical entities of system S, distinct from W.

Neither form of DC can serve as a form of the strong emergence of molecular structure because neither reflects the idea that the structure of a molecule causally produces lower-level effects on its *own* quantum mechanical entities. This is illustrated by contrasting the case under examination with a case that both Kim and Hendry consider as a characteristic example of non-reflexive diachronic DC; namely that of a falling vase (Kim 1999: 26; Hendry 2010b: 189). A vase of 1 kg is thrown towards a window at t_1_, and at t_2_ both the window and the vase break. Assume that one focuses on the relation between the two following systems.

- System W at t_1_: This is described in terms of entities, etc. that are found at the macroscopic level, and it includes all and only the entities and properties of the unbroken vase (i.e. its shape, its weight, etc.). Suppose that these macroscopic properties supervene on properties that are found at a microscopic level at t_1_, and also suppose that at least certain of the macroscopic entities possess causal powers at t_1_ that are not token identical to any set of causal powers possessed by the entities on which they supervene.
- System S at t_2_: This is described in terms of entities, etc. that are found at the microscopic level and it includes all and only the molecules and relevant properties of the broken window. Suppose that the properties that describe the broken window at the macroscopic level at t_2_ supervene on the properties of the microscopic level (i.e. the molecules and their properties).

The falling vase produces a number of changes at the molecular level of its surrounding environment and changes the molecular properties of the objects with which it collides (i.e. the window). Assuming that the vase’s macroscopic properties (weight, shape etc) supervene on its molecular properties, and that certain of the vase’s causal powers are not token identical to any set of powers possessed by its molecules at t_1_, then one could argue that this is a case of non-reflexive diachronic DC. This is because, there are certain molecular properties of a distinct system (i.e. system S) that have been produced at t_2_ by the falling of the vase at t_1_ (i.e. system W).

Regardless of whether these assumptions are well supported, the above example illustrates that the case of the structure of a molecule does not fall under the set of cases that are purportedly genuine examples of non-reflexive DC (either diachronic or synchronic). This is because neither of the two notions of DC reflect the idea that the structure of a molecule causally produces lower-level effects on its *own* quantum mechanical entities. Note that this argument is based on the assumption that the molecule at t_2_ is not a distinct system from the molecule at t_1_. This is a justified assumption because the system that is under investigation in Hendry’s account, is a molecule whose chemical properties (including its structure) do not change due to (or during) the exertion of downward causal powers. That is, the system under examination is a stable molecule with unchanging (average) structure.

### Reflexive diachronic DC

There is one last notion of DC that requires examination; reflexive diachronic DC. This is formulated as a form of strong emergence of molecular structure as follows. There is at least one non-empty set of token causal powers:

- possessed by the chemical entities which define system M ‘s structure (namely the structure of a molecule) at t_1_;
- which is not token identical to any set of causal powers possessed by the quantum mechanical entities of M at t_1_; and,
- which determines certain aspects of the quantum mechanical entities of M at t_2_, in the sense that it produces a change in some set of the entities of M of t_1_.

This subsection examines the coherence and tenability of this notion of DC by distinguishing between two sets of exemplary cases of diachronic reflexive DC.^11^ The first set includes examples such as:

- A vase falling and breaking (Kim 1999: 25–26);
- ‘I fall from the ladder and break my arm’ (Kim 1999: 30); and
- ‘I walk to the kitchen for a drink of water and ten seconds later, all my limbs and organs have been displaced from my study to the kitchen’ (Kim 1999: 30).

The second set includes examples such as:

- The structure of a molecule determines aspects of its quantum mechanical behaviour;
- ‘a cell constrains what happens to its own constituents’ (Paoletti and Orilia 2017: 1); and
- ‘a body regulates its own processes’ (Paoletti and Orilia 2017: 1).

Concerning the first set, the case of the vase falling and breaking is formulated as follows. Consider a vase of 1 kg which is thrown to a window at t_1_, and at t_2_ breaks. Assume that one focuses on the relation between the two following systems.

- M-at-t_1_: This is described in terms of entities, properties etc. that are found at the macroscopic scale and it includes all and only the entities and properties of the unbroken vase that are relevant to that scale.
- M-at-t_2_: This is described in terms of entities, properties etc. that are found at the microscopic scale and it includes all and only the molecules and relevant properties of the broken vase that are relevant to that scale.

The relation between the two systems could be formulated in terms of a relation between the causal powers possessed by higher-level entities of system M-at-t_1_, and some set of the lower-level entities that describe M-at-t_2_. Assuming that the vase’s macroscopic properties (weight, shape, etc.) supervene on its molecular properties at t_1_, and that certain of the vase’s causal powers are not token identical to any set of powers possessed by its molecules at t_1_, one could argue that this is a case of reflexive diachronic DC.; the causal powers of the macroscopic entities of the falling vase at t_1_ produce changes to its lower-level molecular entities, in such a way that determines aspects of how the molecules behave at the state in which the vase is broken (at t_2_).

Concerning the second set, the case of the molecule could be formulated in terms of diachronic reflexive DC as well. Consider a molecule that is in isolation. The molecule is considered to be stable, and thus, unless some external change is imposed on it (i.e. unless it is no longer isolated), it will remain unchanged in terms of its chemical identity. This implies that, although the molecule undergoes continuous dynamical change at the quantum mechanical scale, there is no change in its chemical identity. In this context, the examined case focuses on the relation between the two following systems.

- M-at-t_1_: This is described in terms of entities, etc. that are found at the chemical scale and it includes all the entities, etc. of the molecule at t_1_.
- M-at-t_2_: This is described in terms of entities, etc. that are found at the quantum mechanical scale and it includes all the quantum mechanical entities, etc. of the molecule at t_2_.

Assuming that the molecule’s chemical entities supervene on its quantum mechanical ones at t_1_, and that certain of the molecule’s causal powers are not token identical to any set of powers possessed by its quantum mechanical entities at t_1_, then one could argue that this is a case of reflexive diachronic DC. That is, the causal powers of the chemical entities of the molecule at t_1_ produce changes to its quantum mechanical entities, in such a way that determines aspects of how the quantum mechanical entities behave at t_2_.

Regarding these two sets of cases, the formulation of both sets in terms of diachronic reflexive DC is coherent. Unlike any notion of synchronic DC, a diachronic notion of causation “allows” for the higher-level causal powers to be instantiated at t_1_, prior to being “exercised” in any way that produces a change at t_2_.

Consider now the notion’s tenability with respect to each set of cases. Concerning the first set, Kim takes them to be cases of unproblematic causal statements (1999: 30). According to Kim, what possesses the causal powers at t_1_ and produces the respective lower-level effects at t_2_, are the lower-level entities of the system at t_1_ on which the higher-level entities supervene. While he accepts the formulation of statements such as the ‘falling vase at t_1_ caused its molecules to scatter at t_2_’, he suggests that such statements signify the existence of a genuine causal relation *only* at the lower-level of ontology, and *not* a downward causal one. Put differently, he denies that these are genuine cases of diachronic reflexive DC because, unlike how diachronic reflexive DC is defined, there are no causal powers possessed by the higher-level entities that are not token identical to the causal powers possessed by lower-level entities.^12^

Kim states that most emergentists would agree with such an interpretation of causal statements regarding the first set of cases (1999: 31). This is also accepted by Hendry (2010b: 189). However, Hendry denies that the second set of cases are not genuine instances of downward causation. This is because, according to Hendry, in these instances the higher-level entities possess causal powers that are:

(i) novel; and,
(ii) causally relevant to the production of lower-level effects.^13^

Hendry formulates a ‘counternomic criterion for downward causation’ in order to support this claim (2010b: 189). According to this criterion, ‘a system exhibits downward causation if its behaviour would be different were it determined by the more basic laws governing the stuff of which it is made’ (Hendry 2010b: 189). He claims this criterion is satisfied in the case of molecular structure given how quantum mechanics describes it, which is as follows:

i A molecule is described by the Coulombic Schrödinger equation which is constructed by employing the so-called ‘resultant Hamiltonian’ (Hendry 2010a: 210–211, 213). The resultant Hamiltonian takes into account *all* the intra-molecular interactions, and it is constructed using as input *only* fundamental physical interactions and the value of the physical properties of the entities (i.e. masses, charges, etc.) (Hendry 2010a: 208, 212).
ii A resultant Hamiltonian is in practice never used for the solution of the Schrödinger equation. This is primarily due to the equation’s mathematical complexity. Nevertheless, if the Coulombic Schrödinger equation were to be solved, it would not distinguish between different molecular structures (specifically that of isomers), and it would not explain the symmetry properties of molecules (Hendry 2010a: 214; Hendry 2017: 153).^14^
iii Instead, quantum explanations of molecular structure are based on the construction of ‘configurational Hamiltonians’ for the solution of the Schrödinger equation of a molecule (Hendry 2010a: 210–211).
iv Configurational Hamiltonians are constructed on the basis of ad hoc assumptions which impose on the Schrödinger equation the molecular structure that is supposed to be derived from that equation (Hendry 2010b: 186).
v This situation satisfies the counternomic criterion: we did not recover a molecule’s ‘structure from the “resultant” Hamiltonian, given the charges and masses of the various electrons and nuclei; rather we viewed the motions of those electrons and nuclei as constrained by the molecule of which they are part’ (Hendry 2006: 183).

The counternomic criterion implies that it is in principle impossible to specify all the properties of a system based solely on its quantum mechanical description. If this is taken to support DC, then it must be assumed that there are certain properties which do not exist at the scale describable by quantum mechanics. The molecule’s structure should be taken to be in principle impossible to specify quantum mechanically, and thus does not exist at the relevant scale. As Hendry states:

Molecular structures cannot be recovered from the Coulomb Schrödinger equations, but not because of any mathematical intractability. The problem is that they are not there to begin with. (2010b: 186)

This metaphysical interpretation of the nature of molecular structure is based on a very subtle, but important aspect to Hendry’s reasoning. Hendry argues that while quantum mechanics (in the form of the Coulombic Schrödinger equation) specifies all the entities, etc. of a system (at the quantum mechanical scale and at a particular point in time), quantum mechanics is not a ‘theory of everything’. Hendry states this as follows:

non-relativistic quantum mechanics is assumed to be a sort of “theory of everything” for the motions of electrons and nuclei, and therefore for any molecule. Physicists and philosophers (..) usually mean a theory that could- in principle- explain everything that happens in a system to which it is applied, to the extent that it can be explained. Think of Newton’s laws applied to the planetary motions: natural philosophers since Newton’s time have imaged a God’s-eyes-view application of his laws which could be used to predict all future planetary positions, if only we had accurate enough access to their current positions and momenta, plus large enough computers to cope with very detailed and accurate mathematical models of the solar system. (…) The question of whether molecular structure is strongly emergent is, I think, best understood as the question of whether we have good reasons to think that, from a God’s-eye-view, non-relativistic quantum mechanics is a “theory of everything” in this sense, or whether some looser relationship between the dynamics and the evolution of the system is better supported. (2017: 153)

Put differently, quantum mechanics is not a ‘theory of everything’ in the sense that the quantum mechanical description of a system at t_1_ cannot in principle specify its quantum mechanical description at a later time t_2_. This is because the system possesses entities, properties, etc. at t_1_ that are found at a different scale. These entities, in virtue of existing at a distinct scale, cannot be recovered within a lower-level description. Nevertheless, they partially determine how the lower-level entities will behave at a later time. Therefore, the quantum mechanical description of the system at t_1_ is not nomologically sufficient to specify how the system is at t_2_. With regard to a molecule’s structure, this does not merely mean that the quantum mechanical description (via the resultant Hamiltonian) cannot specify the system’s structure at t_1_; rather there is no structure at the quantum mechanical scale at t_1_. The system possesses structure only at the chemical scale and, as such, it partially determines the entities, etc. found at the quantum mechanical scale at t_2_.

Given the above, Hendry’s account of strong emergence is incompatible with the ‘causal closure, or completeness of the physical’ (CCP). The CCP states that ‘physical effects are brought about solely by physical causes via physical laws’ (Hendry 2010b: 185). Hendry rejects the CCP because it is empirically undermined by the fact that the Coulombic Schrödinger equation, if solved, would not distinguish between different molecular structures and it would not explain the symmetry properties of an isolated molecule.^15^

The following section presents the examples from chemistry which Hendry takes to support downward causation empirically.

## The empirical support for strong emergence

The first example concerns the quantum mechanical description of isomers. ‘Isomers’ refers to any set of molecules that contain the same number and kind of atoms, but whose atoms are arranged differently. Isomers have distinct chemical descriptions, and their postulation is vital for the explanation of a variety of physical and chemical phenomena.

If one describes an isomer via the use of its resultant Hamiltonian, then the Schrödinger equation is identical with the Schrödinger equations that describe the other relevant isomers (Hendry 2017: 153). On the other hand, if one is to describe an isomer via the use of its configurational Hamiltonian, then the Schrödinger equation that is subsequently constructed, is not identical to those that describe the other relevant isomers. According to Hendry, this example satisfies the counternomic criterion. It illustrates that the molecule’s behaviour as this is described ‘by the more basic laws governing the stuff of which it is made’ (i.e. via the resultant Hamiltonian), is different from its behaviour as this is described by assuming certain chemical properties (namely its structure) via the configurational Hamiltonian.

The second example that Hendry takes as empirical support for DC concerns the symmetry properties of molecules. Similarly to the case of isomers, one cannot derive the different chemical symmetry properties from the quantum mechanical description:

the only force appearing in molecular Schrödinger equations is the electrostatic or Coulomb force: other forces are negligible at the relevant scales. But the Coulomb force has spherical symmetry. How, from this slim basis, do we get the great variety of different symmetry properties (chiral (asymmetrical), cylindrical, hexagonal and many more) exhibited in real molecules? (Hendry 2017: 154)

Both examples are taken by Hendry to satisfy the counternomic criterion and thus empirically support the strong emergence of molecular structure.

## Challenges to the strong emergence of molecular structure

This section presents three challenges to Hendry’s account of strong emergence that have remained unnoticed in the literature. Identifying and resolving these challenges is essential for rendering Hendry’s account a viable and convincing position about the nature of molecular structure as a strongly emergent property.

### Incoherence with respect to Supervenience

First, there is an incoherence between one of the main tenets of strong emergence (namely supervenience) and the empirical support of the strong emergence of molecular structure. For Hendry, DC is supported by how the Coulombic Schrödinger equation (via the resultant Hamiltonian) describes a molecule. He disregards the success of the configurational Hamiltonian in distinguishing molecules in terms of their structure. This is because, ‘(w)ithout a quantum-mechanical justification for the attributions of structure (and the lower symmetry)’ configurational Hamiltonians ‘simply assume the facts about molecular structure that ought to be explained’ (Hendry 2010b:186).

This leads to incoherence in Hendry’s account, because, it equally undermines the grounds for thinking supervenience holds, which is one of the core elements of Hendry’s understanding of strong emergence. Consider, for example, the two isomers ethanol and methoxymethane; they are chemically distinct and thus have different chemical descriptions (Hendry 2010a: 214). The resultant Hamiltonians, and thus the Coulombic Schrödinger equations which quantum mechanically describe the two isomers, are the same. Therefore, ethanol and methoxymethane have different higher-level descriptions, whereas their respective lower-level descriptions (as specified by the Coulombic Schrödinger equation) are the same. In sum, if as Hendry recommends, we disregard a molecule’s configurational Hamiltonian, then it follows that there are molecules whose chemical descriptions are different, whereas their quantum mechanical descriptions are the same. So, supervenience would not hold.

Based on the above, one needs to justify whether and why the configurational Hamiltonian is used as putative empirical evidence for supervenience. If one accepts the use of the configurational Hamiltonian for the empirical support of supervenience, then one needs to justify why the configurational Hamiltonian can be used as putative empirical evidence for supervenience, but not as putative empirical evidence for the existence of structure at the quantum mechanical scale. On the other hand, if one argues that the configurational Hamiltonian of a molecule cannot be invoked for the empirical support of supervenience, then one needs to explain why, despite the fact that the resultant Hamiltonian describes different isomers in an identical manner, supervenience is accepted within one’s understanding of strong emergence.

### The assumption of determinate nuclear positions

A second problem in the defence of strong emergence concerns the assumption of determinate nuclear positions.^16^ Recall that quantum mechanics standardly describes a molecule’s structure via the configurational Hamiltonian which, unlike the resultant Hamiltonian, makes assumptions about the structure of the examined molecule (see subsection 2.3). Among those assumptions is that the nuclei that comprise the molecule hold determinate positions.^17^ The fact that quantum mechanics makes this assumption in order to explain molecules (and their structure) constitutes putative evidence for the strong emergence of molecular structure.

This section shows that there are three plausible and alternative ways that one can interpret Hendry’s claim that the need for this assumption supports strong emergence:

1. If quantum mechanical explanations are conditioned on assumptions that are not derivable from quantum mechanics, then this constitutes evidence for strong emergence.
2. If quantum mechanical explanations are conditioned on assumptions that are based on results from or are justified by higher-level theories, then this constitutes evidence for strong emergence.
3. If quantum mechanical explanations are conditioned on assumptions that are ad hoc, then this constitutes evidence for strong emergence.

In Hendry’s work on strong emergence there are passages which are suggestive of each of these three interpretations; the remainder of this section demonstrates this by means of textual evidence.^18^ In this way, this section shows that it is not clear how the assumption is supposed to support strong emergence. Moreover, this section evaluates the tenability of each interpretation and discusses potential challenges that could be raised against them. It is concluded that, in its present form, Hendry’s account has not convincingly shown how the assumption of determinate nuclear positions supports the strong emergence of molecular structure.

#### Interpretation (1): If quantum mechanical explanations are conditioned on assumptions that are not derivable from quantum mechanics, then this constitutes evidence for strong emergence.

The following passage suggests that Hendry has in mind something along the lines of interpretation (1):

The complaint is not that there are no explanations of empirically determined molecular shapes, or even that the explanations are ad hoc, or of poor quality. Rather it is that *the explanation is conditioned on determinate nuclear positions*: if electronic motions are constrained by a stable nuclear backbone, then the energy dependence is such that such-and-such is the lowest energy configuration. (Hendry 2006: 184-185)^19^

According to this interpretation, assuming determinate nuclear positions shows that quantum mechanics is not otherwise sufficient to derive an explanation of the examined molecule. That is, quantum mechanics underdetermines facts about molecular structure. This indeed undermines intertheoretic reduction to the extent that the latter requires lower-level theories to derive the properties of the examined system from the bottom-up.

Moreover, if the purported inability of quantum mechanics to derive molecular structure is not solely understood as an in-practice feature of quantum mechanics, then strong emergence may be reinforced. Indeed, as mentioned in 2.3, Hendry takes quantum mechanics to be *in principle* unable to derive molecular structure; it is not just the case that molecular structure is not derived by quantum mechanics, but that it is not *derivable* by it.^20^ This is a more serious problem in the sense that it could also challenge non-reductive physicalism and thus further support strong emergence (Hendry 2010a: 212–213).

Nevertheless, if this is how Hendry invokes the use of this assumption in quantum mechanics, then more has to be said about why quantum mechanics is in principle unable to derive molecular structure. Stating that this inability is simply due to the fact that molecular structures ‘are not there to begin with’, is not sufficient (Hendry 2010b: 186). This is because, even though quantum mechanics (in the form of resultant Hamiltonians) does not identify the particular structure a molecule is observed to have, it does specify the possible structures of the molecule in the form of superposition states (Claverie and Diner 1980: 59; Scerri 2012; Woolley 1998). Hendry admits this:

The spherically symmetrical states could perhaps be regarded as superpositions of asymmetrical states with opposite orientations, just as the spin states of a silver atom may be regarded as superpositions of spin-up and spin-down, or the quantum state of Schrödinger's cat can be regarded as a superposition of ‘cat-alive’ and ‘cat-dead’ states. (2010a: 214)

In fact, there is a close connection between the putative empirical evidence for strong emergence and foundational problems in quantum mechanics. Scerri illuminates this connection quite clearly for the case of isomers:

According to quantum mechanics, molecules can be said to be in a superposition of a number of possible structures. For example, C_2_H_6_O_1_ can be regarded as a superposition of quantum states representing the structures of quantum states representing the structures of C_2_H_5_OH and CH_3_OCH_3_. Woolley, and now Hendry, concentrates on the fact that until an observation is carried out, neither of these structures has been actualized. The inference they draw from this state of affairs is that there is no intrinsic structure in the molecule, which—if it were true—would indeed mean that structure is not fundamental. However, the study of decoherence has shown that it is not just observations that serve to collapse the superpositions in the quantum mechanical equations. The collapse can also be brought about by the molecules interacting with their environment, something that Hendry occasionally mentions but quickly dismisses.^21^ (Scerri 2012: 23)

Scerri’s formulation of Hendry’s argument suggests that the inability of resultant Hamiltonians to describe molecular structure is based on the fact that they identify superpositions of quantum states with alternative corresponding molecular structures. From this it follows that quantum mechanics’ *in principle* inability to identify structure is conditioned on some understanding of superpositions, as well as of the collapse of the wavefunction (also referred to as the measurement problem (Cushing 1998: 309)).

However, how to interpret superposition states and the collapse of the wavefunction are not settled issues within science and philosophy, and various proposals have been offered to explain them (Myrvold 2018). Given this, one cannot simply assume that a quantum mechanical description of molecular structure in terms of superposition states is not a description which adequately explains a molecule’s observed structure. Given that different metaphysical understandings of superposition states are debated in the literature, the strong emergentist needs to justify how she understands superposition states and why such states do not in principle explain structure.

Of course, establishing such a connection between the putative evidence for strong emergence and these foundational problems in quantum mechanics requires a much more detailed analysis that this paper cannot offer.^22^ Nevertheless, it is interesting to note that other philosophers of chemistry also connect quantum mechanics’ inability to identify structure with foundational problems in quantum mechanics (see Chang 2015; Lombardi and Castagnino 2010). If Hendry’s argument in favour of strong emergence is similarly based on considerations of foundational problems in quantum mechanics, then a lot more has to be said regarding how the strong emergentist interprets superpositions and deals with the measurement problem. If, on the other hand, Hendry does not assume a connection with foundational problems in quantum mechanics then this interpretation seems to be lacking sufficient empirical evidence as it is no longer clear on what grounds quantum mechanics (in the form of resultant Hamiltonians) is in principle unable to identify structure.

In sum, a lot more has to be said regarding quantum mechanics’ inability to identify structure in order to invoke this as support for strong emergence. If quantum mechanics’ inability to identify structure is based on the fact that it identifies superpositions of molecular structures, then a central question that Hendry needs to address is how superposition and collapse are interpreted. This, in turn, leads to questions about the classical-quantum divide, the measurement problem and its standard putative solutions. None of these issues have been considered by Hendry in the context of evaluating the strong emergence of molecular structure.^23^

All in all, while this interpretation of Hendry’s argument holds significant strength, there is an important feature missing for the argument for strong emergence to be sufficient. In the context of this interpretation, quantum mechanics’ inability to derive molecular structure is connected to foundational issues in quantum mechanics. This connection should be brought into light and relevant views on these issues should be taken into account in order to convincingly claim that molecular structure strongly emerges.

#### Interpretation (2): If quantum mechanical explanations are conditioned on assumptions that are based on results from or are justified by higher-level theories, then this constitutes evidence for strong emergence.

Hendry states the following which is suggestive of interpretation (2):

To solve the Schrodinger equations for more complex atoms, or for any molecule, quantum chemists apply a battery of approximate methods and models. Whether they address the electronic structure of atoms or the structure and bonding of molecules, these approximate models are calibrated by an array of theoretical assumptions *many of which are drawn from chemistry itself.* (Hendry 2010a: 212)^24^

That this is a sensible interpretation of Hendry’s argument is also supported by the following passage:

To invoke one important constituency, among the chemists who founded quantum chemistry, there were many temperamental non-reductionists, who saw the quantum-mechanical explanation of chemical structure and bonding as a process that drew equally of physical principles and chemical knowledge, adapting quantum mechanics significantly in the process. (2010b: 187)

On this interpretation of Hendry’s argument, strong emergence is supported because the assumption of determinate nuclear positions is in some sense based on results from or justified by chemistry. Prima facie, this suffices to support that molecular structure strongly emerges at the chemical scale; if the assumption which is built into the quantum mechanical formalism somehow involves reference to chemical results or justifications (especially about molecular structure), then this could motivate the claim that molecular structure strongly emerges. However, there are no sufficient grounds to claim that this assumption is based on results from or justified by chemistry.

First, the assumption of determinate nuclear positions is formulated exclusively in terms of lower-level (i.e. quantum mechanical) entities, properties, etc. The entities, etc. that are invoked for the application of this assumption are physical. No chemical entity or property is invoked for the application of this assumption to the Schrödinger equation. This is evident by how the BO approximation is defined:

Representation of the complete wavefunction as a product of an electronic and nuclear part (Ψ ( r, R ) = Ψ_e_( r,R ) Ψ_Ν_( R )) where the two wave-functions may be determined separately by solving two different Schroedinger equations. The validity of the Born–Oppenheimer approximation is founded on the fact that the ratio of electronic to nuclear mass (…) is sufficiently small and the nuclei, as compared to the rapidly moving electrons, appear to be fixed. The approximation breaks down near a point where two electronic states acquire the same energy. The BO approximation is often considered as being synonymous with the adiabatic approximation. More precisely, the latter term denotes the case when Ψ_e_ diagonalize the electronic Hamiltonian. Thus, the adiabatic approximation is an application of the BO approximation. (IUPAC 2014: 179)

Secondly, assuming determinate nuclear positions does not amount to assuming a higher-level property, namely molecular structure. That nuclei hold determinate positions does not imply that the entire molecule has a particular structure. This is because a given set of nuclear positions corresponds to more than one structure. Each set of nuclear positions is compatible with different quantum states of the system because the electrons behave in more than one possible way, thus resulting in more than one structure. Quantum mechanics itself specifies which quantum state of the molecule (and thus which structure) corresponds to the stable state of the molecule.^25^ Therefore, the conclusions drawn from quantum mechanics about how a molecule is structured, are not presupposed by assuming determinate nuclear positions.^26^

Given the above, the only reasonable way this assumption can support strong emergence in the context of interpretation (2) is that it somehow appeals to higher-level (i.e. chemical) facts.^27^ In order to illustrate this, consider how this assumption is applied to quantum mechanics. Within the BO approximation, one can in principle formulate the Hamiltonian operator by positioning the nuclei at all the possible determinate positions. Each set of nucleonic positions corresponds to different quantum states of the system (hence to different wavefunctions) and to different values of the total energy, E, of the molecule. However, in practice this process is not followed. By having prior knowledge of the quantum system that is under examination (in this case, by knowing the chemical properties of a molecule), only particular nucleonic conformations are considered when constructing the Hamiltonian operator. It is only in this sense that the assumption of determinate nuclei positions may be considered to be based on results from chemistry. This however is an epistemic feature of quantum mechanics; it concerns how the assumption is applied to the Schrödinger equation. In principle, it is possible to apply this assumption by considering all possible sets of nucleonic positions and thus without invoking chemical facts about the examined system (Atkins 1974: 29).

Given all the above, unless one proposes a different way to cash out interpretation (2), this interpretation of Hendry’s argument is not a convincing way of defending the strong emergence of molecular structure.

#### Interpretation (3): If quantum mechanical explanations are conditioned on assumptions that are ad hoc, then this constitutes evidence for strong emergence.

Lastly, while Hendry states that ad hoc-ness is not the source of his ‘complaint’ against reductionism, there are other parts where he suggests that the use of ad hoc assumptions is in fact problematic^28^:

The Coulomb Schrödinger equation for an n-molecule ensemble of hydrogen chloride molecules has precisely the same symmetry properties as a Coulomb Schrödinger equation for a 1-molecule system. If the particular form of the symmetry-breaking addition is not justified, *then it is just ad hoc: a deus ex machina.* (Hendry 2010a: 215)^29^

This quote prompts the investigation of the use of ad hoc assumptions and how they should be evaluated when it comes to the investigation of metaphysical positions such as strong emergence. There is extensive literature about what constitutes a hypothesis ad hoc, and in what instances it is unacceptable for the theory that assumes it. Without analyzing it in detail, certain points can be drawn from the relevant literature. Specifically, it is argued that while the particular assumption is a hypothesis which is neither derived nor required by the theory in which it is assumed, it is not ad hoc in the sense of being an ‘unacceptable’, ‘arbitrary’, ‘contrived’, or ‘strange’ hypothesis (Hunt 2012: 11).

First, according to Hunt, if ‘an auxiliary hypothesis turns out to make a novel prediction about which the progenitor of the idea was not aware’, it should not be considered ad hoc in the unacceptable sense of the term (Hunt 2012: 3; see also Grünbaum 1964: 1409). Indeed, the addition of this assumption allows the quantum mechanical description to make novel predictions about a molecule’s structure. These predictions have not only contributed to the empirical verification of the theory, but also to the formulation of novel explanations about molecular structure. Quantum mechanics has revealed the effect of electron delocalisation on the overall stability of a molecule’s structure (Weisberg 2008: 939–943); it has specified atomic structures and led to the prediction of novel elements (Needham 2004: 213); and quantum models have made novel predictions about, among others, pericyclic reactions (Hendry 2004: 1057), large molecules, and metals. This undermines the claim that this is an ‘unacceptable’ assumption to make in quantum mechanics.

Secondly, it is important to take into account the role and prevalence of ad hoc assumptions in science, when evaluating metaphysical claims which are supported via those scientific practices. Quantum mechanics more often than not employs information from classical physics or the special sciences in order to describe some phenomena. Depending on the particular system which it is set to describe, quantum mechanics draws information from the relevant classical or special science theories, in order to derive the properties of the system in a manner consonant to experimental evidence. An example is the case of wavefunction scarring, as discussed by Bokulich (2008a). She examines how semiclassical mechanics explains quantum phenomena in classical systems which are chaotic (2008a: 219). Similarly to the case of molecular structure, the explanation of the behaviour of quantum systems is provided by reference to classical structures. This is allegedly problematic because ‘the classical structure that they appeal to do not, strictly speaking, exist in these quantum systems’ (Bokulich 2008a: 217). Another example concerns quantum electrodynamics. In this context, ‘(i)t has become conventional to interpret experiments on the electromagnetic properties of leptons in terms of ad hoc modifications introduced in the formulas of quantum electrodynamics’ (Kroll 1966: 65).

In light of the above, the supporter of strong emergence could say that these are further examples of strong emergence. On this view, it is not only molecular structure which strongly emerges; various properties of a system, as these are described by classical physics or the special sciences, strongly emerge from its quantum mechanical behaviour. On the other hand, one could say that the prevalence of such examples indicates that something not so special is going on; this is merely how scientific explanations are derived from our theories. For example, Bokulich argues that while a purely quantum mechanical explanation of the phenomenon (i.e. ‘without reference to classical structures’) is ‘in some sense deficient’, ‘the classical trajectories do not cause the scarring’ (2008a: 219 and 228). If strong emergence is to be supported by the ad hoc assumptions about molecular structure, then the overall use of ad hoc assumptions in quantum mechanics has to be addressed.

One last point against the idea that the use of ad hoc assumptions gives empirical support to strong emergence, concerns the role of ad hoc assumptions in the construction of a molecule’s resultant Hamiltonian. Hendry’s argument for strong emergence assumes that the Coulombic Schrödinger equation (via the resultant Hamiltonian) makes no prior assumptions about the system it describes, and it is based only on the main postulates of quantum mechanics (Hendry 2010a: 212–213). However, this is not the case. The construction of the Coulombic Schrödinger equation is based on prior knowledge of the behaviour of particles. For example, the fact that the equation is spherically symmetric is something that is not derived from basic principles of physics but is an ad hoc assumption that is made in order to formulate the equation. Therefore, if one understands Hendry’s argument as being based on the use of ad hoc assumptions, then one must conclude that strong emergence is untenable because the spherical symmetry of the Coulombic Schrödinger equation is an ad hoc assumption as well.

This concludes the examination of the possible ways that Hendry’s argument can be interpreted. Each interpretation is vulnerable against particular challenges that potentially undermine the support of strong emergence. So it is crucial that one spells out exactly how the assumption of determinate nuclear positions supports strong emergence.

### The nature of causation in downward causation

So far, this paper assumed an understanding of causation in terms of a primitive notion of production. It pointed out that Hendry does not explicitly state which notion of causation he assumes when referring to DC. Nevertheless, Hendry presents a counternomic criterion which, according to him, sufficiently supports DC. Specifically, Hendry takes that this criterion supports DC because the quantum mechanical description is nomologically sufficient for specifying the structure of a molecule, only after the use of ad hoc assumptions. Put differently, DC is understood by Hendry as a relation of nomological sufficiency.

The problem with such an understanding of DC is that, even if the quantum mechanical system at t_1_ is not nomologically sufficient for the occurrence of the quantum mechanical system at t_2_, this does not necessarily imply that the chemical system at t_1_ determines the quantum mechanical system at t_2_. For DC to be tenable, it must also be argued that the chemical entities, etc. at t_1_ are nomologically sufficient for the determination of the quantum mechanical entities, etc. at t_2_. That is, the chemical system must go through the same level of scrutiny that the quantum mechanical system at t_1_ goes through and allegedly fails. For that to be the case, it must be that the chemical description *alone* is nomologically sufficient for the occurrence of the quantum mechanical system at t_2_.^30^

This subsection argues that the chemical entities, etc. at t_1_ are not nomologically sufficient for the determination of the quantum mechanical entities, etc. at t_2_. It introduces Hall’s account of ‘causation as production’ and argues that, within Hall’s account, the chemical entities, etc. are not nomologically sufficient at t_1_ for the determination of the quantum mechanical ones at t_2_ (Hall 2004).

According to Hall, a system M, understood as a set of all the entities, properties, processes, etc. that define M, sufficiently produces a system M’, just in case M’ follows from:

(i) the laws;
(ii) the premise that all the members of M occur at t_1_; and,
(iii) the premise that no other events occur at t_1_.^31^

Hall does not examine ‘causation as production’ with respect to systems that are described within different scales, so this subsection makes three clarifications. First, since the description of all systems is scale dependent, the laws that are employed in order to describe each system must include all and only those concepts, theoretical postulations, explanatory mechanisms and laws of nature that are relevant to that specific scale. Secondly, the members of M are taken to include all and only those entities, properties, causal powers, etc. which completely specify the system at the relevant scale.^32^ That is, neither the laws nor the entities, properties, etc. should belong to a different scale from that in which the respective system is specified. Thirdly, in the case under examination, there are two systems that are taken to be causally related via diachronic reflexive DC. The first system is that of an isolated molecule at t_1_ (i.e. system M). M is the set of all and only those chemical entities, properties, causal powers, etc. that occur at t_1_ and specify the molecule.^33^ The second system M’ occurs at t_2_, and is the set of the quantum mechanical entities, properties, etc. at t_2_. Notice that the change that has been produced to the quantum mechanical entities, etc. from t_1_ to t_2_ has not led to a change in the chemical properties of the molecule at t_2_. The structural properties of the molecule produce changes to certain of its quantum mechanical properties in such a way that does not change those structural properties. Put differently, the structure of the entire molecule produces certain aspects of the behaviour of the electrons and nuclei in such a way that maintains this structure.

In order for M to causally produce a reflexive diachronic change, it must be the case that the set M, in accordance with the chemical laws, are sufficient for a change at the quantum mechanical level to occur at t_2_. This must follow from:

(i) the chemical laws;
(ii) all the members of M that occur at t_1_; and,
(iii) no other events outside of M occurring at t_1_.

However, the chemical laws, together with the set M at t_1_, are not sufficient for determining the quantum mechanical description of the system at t_2_. This is for two reasons. First, it is not possible to derive the quantum mechanical description of system M’ at t_2_ from the chemical description of system M at t_1_, and the relevant chemical laws. The chemical laws and chemical entities, etc. cannot derive the sort of fine-grained description required for the full specification of the quantum mechanical system at t_2_. Therefore, this model of production collapses and, consequently, diachronic reflexive DC in terms of production is not epistemically supported.

A possible reply to this objection is that, although it is not possible to derive the quantum mechanical description of the system at t_2_ from the chemical description, laws, etc. of the system at t_1_, this doesn’t preclude the possibility that chemical systems cause quantum mechanical changes. This reply to the first objection leads to the second objection against DC. Due to multiple realisability, it is possible that a molecule, as this is described in chemistry, supervenes on more than one quantum mechanical state. Put differently, there are more than one possible quantum mechanical states of M’ on which the molecule (both at t_1_ and at t_2_) supervenes. In light of this, it is not possible that the chemical system alone, without ‘taking into account’ the particular lower-level entities that occur ‘within’ it at that particular time, can determine the state of its lower-level entities, etc..

A possible reply to this objection is that the set M includes all possible members of the basis on which M supervenes, and not only the actual set of entities, properties, etc. on which the molecule supervenes at that particular time. If M is understood this way, then the set M suffices for the production of the quantum mechanical change.^34^ However, this would not be equivalent to stating that a molecule at t_1_ produces quantum mechanical changes on its supervenience basis at t_2_, but rather that the molecule together with its supervenience basis at t_1_ produce such changes at t_2_.

Based on the above analysis, the chemical system at t_1_ is not nomologically sufficient for the production of the quantum mechanical system at t_2_. It should be noted that, in the context of Hall’s account, the configurational Hamiltonian and its respective Schrödinger equation cannot be regarded as the higher-level description that supports the nomological sufficiency of the chemical system. The configurational Hamiltonian that is used for formulating the quantum mechanical description of a molecule, is neither a chemical description of that molecule at t_1_, nor is it based on the application of chemical laws pertaining to the molecule. Indeed, some assumptions and approximations that are used in order to construct the configurational Hamiltonian have been based on the respective chemical description of the system at t_1_. However, the configurational Hamiltonian is still part of a quantum mechanical description because the entities, properties, etc. that are postulated, as well as the interactions that are described, are found at the quantum mechanical scale.

A possible way out for the supporter of strong emergence is to propose a different notion of causation. This however requires a detailed presentation of how the proposed notion of causation supports the metaphysical claims that (1) the quantum mechanical entities, etc. at t_1_ do not determine certain aspects of the system’s quantum mechanical behaviour at t_2_, and *also* that (2) the chemical entities, etc. at t_1_ determine certain aspects of the system’s quantum mechanical behaviour at t_2_.

## Conclusion

This paper argues that Hendry’s account of strong emergence is coherent only under a notion of reflexive diachronic DC. While diachronic reflexive DC is the most appropriate candidate for the case under examination, there are three challenges to the strong emergence of molecular structure that have not been identified in the literature. First, the empirical evidence presented for strong emergence equally undermines supervenience. Secondly, it is ambiguous how the assumption of determinate nuclear positions is invoked for the support of strong emergence as this feature of Hendry’s argument can be interpreted in more than one ways. Lastly, there are understandings of causation which render the postulation of a downward causal relation between a molecule’s structure and its quantum mechanical entities, untenable.

Depending on one’s metaphysical inclinations, these challenges can be viewed as either arguments that undermine the tenability of the strong emergence of molecular structure, or as opportunities to identify and improve upon the weaknesses of this account. In any case, the goal of this paper is to contribute to the discussion about metaphysical emergence and to enhance our understanding of strong emergence in chemistry.

## Footnotes

1 The term ‘weak emergence’ is also sometimes taken to refer to a metaphysical position. As Baysan and Wilson state, ‘accounts of metaphysical emergence (…) are typically characterized as either “weak” or “strong”, reflecting whether or not the emergence at issue is supposed to be compatible with physicalism’ (2017: 51).

2 An exception is Humphreys who formulates a taxonomy of emergence that doesn’t involve the distinction between epistemic and metaphysical emergence (2016).

3 Obviously, there may be additional kinds of disagreements that are not mentioned here.

4 This paper leaves open whether alternative metaphysical accounts of strong (or weak) emergence could successfully be defended with respect to molecular structure.

5 This paper’s formulation of Hendry’s account makes reference to other philosophers that have also examined accounts of strong emergence (namely Humphreys 2016; Kim 1999, 2006; Wilson 2015). Their understanding of strong emergence is compatible with Hendry’s account and contributes to a deeper understanding of the strong emergence of molecular structure.

6 Humphreys, for example, examines the possibility of there being no levels (2016).

7 See, for example, the discussion of levels in terms of mechanisms in (Bechtel 2017) and (Eronen 2013). Also, note that this paper does not examine whether there is a final, fundamental lower level (for example see Ladyman et al. 2007).

8 This understanding of levels is consistent with Hendry’s formulation, so alternative understandings are not considered here.

9 This paper doesn’t examine what sort of things possess causal powers. For simplicity, it mentions entities and properties as the possessors of causal powers. The paper understands powers in a ‘neutral’ manner, ‘reflecting commitment just to the plausible thesis that what causes an entity may potentially bring about are associated with how the entity is’ (Wilson 2015: 348). Nevertheless, the nature of causation in strong emergence is evaluated later on. Also, note that the acceptance of the causal autonomy of the higher-level, together with the postulation of a dependence relation, is allegedly problematic if one also accepts the Causal Closure of the Physical (CCP). This issue is referred to as the overdetermination problem and is not discussed here.

10 Italics are added here.

11 The first set of examples is examined by Kim in relation to diachronic reflexive DC. Regarding the second set, as mentioned in section 2 it is not clear which particular notion of DC Hendry assumes with respect to molecular structure. Whether a similar ambiguity exists in the literature concerning the other examples of the second set, is not currently considered. Instead, it is assumed here that, since the second set of examples have been regarded as being similar to that of the case of molecular structure, and since the other notions of DC have been rejected in the previous subsections, then the only remaining alternative is to consider these examples in terms of diachronic reflexive DC.

12 Kim’s point challenges strong emergence because he denies the causal autonomy of the higher-level.

13 This paper assumes that higher-level causal powers are novel if they are not token identical to any set of causal powers possessed by the respective lower-level properties on which the higher-level properties supervene. Moreover, as already noted in section 2, it is not clear whether Hendry takes higher-level entities to be causally necessary or sufficient for the production of lower-level effects (hence the use of the term ‘causally relevant’ in clause (ii) above- see also subsection 4.3).

14 According to Hendry, clauses (i) and (ii) together support the rejection of the ‘causal closure, or completeness of the physical’ (Hendry 2010a: 210, 216). This feature of Hendry’s account is briefly presented below.

15 Hendry proposes an alternative universality principle; namely the ‘ubiquity of physics’ (UP). UP allows for the physical principles (as these are formulated via the physical laws and theories) to ‘apply universally without accepting that they fully determine the motions of the systems they govern’ (Hendry 2010b: 188). Hendry’s rejection of the CCP and support of the UP are not further examined here.

16 Special thanks to the reviewers for their helpful and insightful comments on this section.

17 This assumption is applied to the Schrödinger equation via the Born-Oppenheimer approximation which constructs a “(r)epresentation of the complete wavefunction as a product of an electronic and nuclear part” (IUPAC 2014: 179).

18 I do not exclude the possibility that there are other interpretations that one could offer (see more details below). Nevertheless, given that this paper focuses exclusively on how Hendry presents and supports strong emergence, I consider here only those interpretations for which there is textual evidence from Hendry’s work.

19 Italics are added here.

20 Recall that, according to Hendry, “molecular structures cannot be recovered from the Coulomb Schrödinger equations, but not because of any mathematical intractability. The problem is that they are not there to begin with” (2010b: 186).

21 Note that, similarly to Hendry, Scerri does not consider how superpositions should be interpreted, nor does he take into account any of the standard putative solutions that have been offered in order to explain the collapse of the wavefunction (for example Myrvold 2018).

22 Another paper is currently under preparation that is dealing with this issue in detail.

23 An exception is the issue of decoherence in quantum mechanics which Hendry briefly considers (and subsequently rejects) as a possible objection to strong emergence (2010a: 216).

24 Italics added here.

25 Of course, quantum mechanics’ ability to specify molecular structure in this way is conditioned on the assumption that nuclei hold fixed positions (as Hendry correctly points out). If the latter is supportive of strong emergence, then this brings us back to interpretation (1) which, as shown previously, faces other challenges.

26 Interestingly, Chang (2015) and González et al. (2019) claim that by applying this assumption, nuclei are treated as classical particles as they contravene Heisenberg’s uncertainty principle. They argue that this fact undermines the reduction of chemistry to quantum mechanics. I believe that Chang’s (2015) and González et al.’s (2019) arguments relate to foundational problems in quantum mechanics and in particular to the issue of the classical-quantum divide (see Bokulich 2008b). However, examining Chang’s (2015) and González et al.’s (2019) positions goes beyond the scope of this paper, so I cannot support this view here. Nevertheless, even if one considers such views as plausible interpretations to Hendry’s argument, this further reinforces the need to demystify how the support of strong emergence is related to foundational issues in quantum mechanics (see Interpretation 1). In any case, this paper only focuses on how Hendry argues for strong emergence. To my knowledge, there is no textual evidence of Hendry discussing Heisenberg’s uncertainty principle in his defence of strong emergence; so, there are no sufficient grounds to argue that this is what Hendry means when discussing determinate nuclear positions.

27 I would like to thank the reviewers for pointing this out.

28 Recall that Hendry states: ‘The complaint is not that there are no explanations of empirically determined molecular shapes, or even that the explanations are ad hoc, or of poor quality’ (2006: 184–185). Recall that the ‘Coulomb Schrödinger equation’ is defined by Hendry as the Schrödinger equation which is constructed via the use of the resultant Hamiltonian.

29 Italics added here.

30 This objection can be challenged if strong emergence requires that the chemical description is nomologically necessary, but not sufficient, for the occurrence of the quantum mechanical system. However, the defender of strong emergence needs to explain why the nomological necessity of the higher-level implies the existence of a *downward* causal relation.

31 Certain modifications are made to Hall’s understanding of causation in order to maintain uniformity with the terms employed in the examination of DC. Also, this subsection disregards the fact that Hall’s account has been formulated in accordance with Mackie’s INUS conditions (Mackie 1965). This is an unproblematic aspect of Hall’s account and it is not examined with respect to DC.

32 The same applies for the effect/system M’, regardless of the fact that M is not explicitly defined as a set.

33 This includes its molecular structure.

34 This however requires the inclusion of the quantum mechanical laws that specify the supervenience basis at t_1_.
